# Prothoracicotropic Hormone Acts as a Neuroendocrine Switch between Pupal Diapause and Adult Development

**DOI:** 10.1371/journal.pone.0060824

**Published:** 2013-04-05

**Authors:** Akira Mizoguchi, Shintaro Ohsumi, Katuji Kobayashi, Naoki Okamoto, Nobuto Yamada, Ken Tateishi, Yoshinori Fujimoto, Hiroshi Kataoka

**Affiliations:** 1 Division of Biological Science, Graduate School of Science, Nagoya University, Nagoya, Japan; 2 Department of Integrated Biosciences, Graduate School of Frontier Sciences, The University of Tokyo, Kashiwa, Chiba, Japan; 3 Safety Management Section, National Institute of Agrobiological Science, Tsukuba, Ibaraki, Japan; 4 Department of Chemistry and Materials Science, Tokyo Institute of Technology, Tokyo, Japan; Ghent University, Belgium

## Abstract

Diapause is a programmed developmental arrest that has evolved in a wide variety of organisms and allows them survive unfavorable seasons. This developmental state is particularly common in insects. Based on circumstantial evidence, pupal diapause has been hypothesized to result from a cessation of prothoracicotropic hormone (PTTH) secretion from the brain. Here, we provide direct evidence for this classical hypothesis by determining both the PTTH titer in the hemolymph and the PTTH content in the brain of diapause pupae in the cabbage army moth *Mamestra brassicae*. For this purpose, we cloned the PTTH gene, produced PTTH-specific antibodies, and developed a highly sensitive immunoassay for PTTH. While the hemolymph PTTH titer in non-diapause pupae was maintained at high levels after pupation, the titer in diapause pupae dropped to an undetectable level. In contrast, the PTTH content of the post-pupation brain was higher in diapause animals than in non-diapause animals. These results clearly demonstrate that diapause pupae have sufficient PTTH in their brain, but they do not release it into the hemolymph. Injecting PTTH into diapause pupae immediately after pupation induced adult development, showing that a lack of PTTH is a necessary and sufficient condition for inducing pupal diapause. Most interestingly, in diapause-destined larvae, lower hemolymph titers of PTTH and reduced PTTH gene expression were observed for 4 and 2 days, respectively, prior to pupation. This discovery demonstrates that the diapause program is already manifested in the PTTH neurons as early as the mid final instar stage.

## Introduction

Diapause is a programmed developmental arrest accompanied by markedly reduced metabolic activity that enables organisms to survive adverse seasons and/or to synchronize their reproductive timing [1]–[3]. This adaptive strategy is very common in insects and has long attracted the attention not only of entomologists but also of amateur naturalists. Insects enter diapause at various developmental stages - egg, larva, pupa or adult - but the stage at which diapause occurs is fixed in each species. In many cases, diapause is induced by environmental cues, usually seasonal changes in day length, and is executed through endocrine control of development. The endocrine mechanism of insect diapause bas been most extensively studied for pupal diapause since the mid 20th century, when Carroll Williams proposed that a temporary failure of the secretion of a “brain hormone”, which was primarily described by Stefan Kopeć in 1922 [4] and is known as the first discovered neurosecretory hormone, and the resulting inactivation of the prothoracic glands (PGs), which secrete the molting hormone needed for pupa-adult development, leads to pupal diapause. This hypothesis was based on his elegant tissue ablation/implantation experiments with diapausing pupae of the giant silk moth *Hyalophora cecropia* [5]–[7]. The brain hormone and the molting hormone were later structurally identified and are now termed prothoracicotropic hormone (PTTH) and ecdysteroid, respectively. Many studies have demonstrated very low ecdysteroid titers in the hemolymph of diapausing pupae [8]–[10] and the induction of adult development by the injection of ecdysteroid into the diapausing pupae [11]–[13]. In combination, these experiments establish that the shutdown of ecdysteroid secretion is critical for the initiation and maintenance of pupal diapause. However, it remains unclear how PTTH is involved in the regulation of diapause, although most researchers believe that the shutdown of PTTH secretion after pupal ecdysis results in PG inactivation.

About two decades ago, PTTH was purified from the heads of the silk moth *Bombyx mori,* and its primary structure was determined [14], [15], followed by a successful measurement of its hemolymph titers during larva-pupa-adult development [16]. A large, long-lasting peak in the PTTH titers was detected after pupal ecdysis, suggesting that a PTTH peak leads to pupa-adult development through the continuous stimulation of the PGs. However, because *B. mori* never undergoes pupal diapause under any environmental conditions, the role of PTTH in the regulation of pupal diapause could not be tested in this species. In some insects, including *Manduca sexta* and *Sarcophaga argyrostoma*, the PTTH activity in the brains of diapausing and non-diapausing pupae was measured [9], [17] using an *in vitro* PG assay [18] for comparison. Unexpectedly, the brain extracts from the early diapausing pupae had equivalent or even higher PTTH activity compared to the extracts from the non-diapausing pupae, indicating that diapause is not a consequence of PTTH paucity in the brain. The authors interpret these results to mean that diapause results from the cessation of PTTH release by the brain but not of its synthesis and storage. In contrast, the measurement of PTTH gene expression levels in the brains of *Heliothis virescens* and *Helicoverpa armigera* showed that the gene expression is much lower in diapausing pupae than in non-diapausing pupae [19], [20], suggesting that PTTH production is actually regulated at the transcriptional level. These seemingly opposite conclusions obtained from work in different species have not yet been reconciled. These lines of research have been pursued with the intention of confirming the cessation of PTTH secretion in diapausing pupae and/or of determining the cause of this cessation. In reality, however, no direct evidence has been obtained for the cessation of PTTH secretion following pupal ecdysis.

Clearly, the simplest way to confirm the shutdown of PTTH secretion is to determine the PTTH titers in the hemolymph of diapausing pupae. For this purpose, we have developed a very sensitive assay to measure PTTH levels in the cabbage army moth *Mamestra brassicae*, which undergoes pupal diapause in short-day conditions. Using this technique, we demonstrate here that PTTH secretion does cease after pupal ecdysis in the diapause-destined animals. Most interestingly, the first sign of PTTH neuron activity decline is already detectable at the mid final instar stage. We also demonstrate that the cessation of PTTH secretion is accompanied by the accumulation of the hormone in the pupal brain and by a reduction in the PTTH gene expression during the late final instar stage, suggesting that the previous conflicting findings obtained in different species can be integrated into a single common mechanism regulating pupal diapause induction.

## Materials and Methods

### Animals

Eggs of *M. brassicae* were obtained from a laboratory colony maintained at the National Institute of Agrobiological Sciences, Japan. The larvae were reared on an artificial diet of “Insecta LFS” (Nihon Nosan Kogyo, Yokohama, Japan) at 25°C under a 14 hr light:10 hr dark photoperiod (long-day conditions) or at 23°C under a 10 hr light:14 hr dark photoperiod (short-day conditions). The animals under the long-day and short-day conditions entered pupal diapause at rates of 0% (0/145) and 98.6% (138/140), respectively.

### Molecular Cloning of *MabPTTH*

Total RNA was extracted from 10 pupal brains using TRIzol reagent (Invitrogen). Single-stranded cDNA for PCR was synthesized from the total RNA with Superscript III reverse transcriptase (Invitrogen). PCR was performed using the following degenerate primers:

sense primer, 5′-(A/G)T(G/T)GATTA(C/T)G(A/C)(A/T)AA(C/T)ATGA-3′;

antisense primer, 5′-TA(A/G)TC(C/T)CT(C/G)GT(A/G)CA(C/G/T)A(C/T)ACA-3′.

These degenerate primers were designed from consensus sequences obtained after the alignment of previously determined PTTH cDNA sequences for *H. virescens* (GenBank accession number: AY172671), *H. armigera* (AY286543), *Helicoverpa zea* (AY172670) and *Helicoverpa assulta* (AY780526). The PCR was performed under the following conditions: 94°C for 5 min and 30 cycles of 94°C for 30 s, 45°C for 1 min and 72°C for 1 min, with a final extension step of 7 min at 72°C. The amplified approximately 400-bp fragments were gel purified, cloned into a pGEM-T vector (Promega) and sequenced. The entire sequence of *MabPTTH* was determined by 5′- and 3′-RACE followed by nested PCR, using the SMART RACE cDNA Amplification Kit (Clontech). The products of the 5′ and 3′ nested PCR were sequenced, and the obtained sequence and its deduced amino acid sequence were analyzed and aligned using GENETIX ver. 5.2 (GENETYX). The primers used were as follows:

5′ RACE primer, 5′-GCCAACGCTGATGGGACGATATTCACC-3′;

3′ RACE primer, 5′-CCGGATCCTTCCTGCGCTTGTAACCC-3′;

5′ nested primer, 5′-CCATGCACTGGTATGGCCAGGGGCATGAC-3′;

3′ nested primer, 5′-CTGGGACGAAACACGTTCCCGCAACGCG-3′.

The sequence reported in this paper has been deposited in the GenBank database (accession no. AB748456).

### *In situ* Hybridization

Whole-mount *in situ* hybridization was performed as previously described [21], with DIG-labeled RNA probes synthesized using the obtained 400-bp DNA fragment as a template.

### Expression and Purification of Recombinant *Mab*PTTH

The recombinant *Mab*PTTH was expressed as previously described [22], with some modifications. The isolated *Mab*PTTH cDNA was amplified with restriction enzyme sites, Nde I and BamH I, by PCR and then inserted into pET3b vector. The initial methionine residue is involved in the Nde I restriction site and is fused with the predicted first amino acid residue of the mature *Mab*PTTH, glycine. The construct was then transformed into the *E. coli* strain BL21, and recombinant *Mab*PTTH was induced by 1 mM IPTG for 7 h.

### Antibodies

Mouse monoclonal antibodies (D17 and D18) and a rabbit polyclonal antibody were generated against the recombinant *Mab*PTTH monomer using standard methods. These antibodies were purified using Protein A affinity chromatography and stored at a concentration of 1 mg/ml. The same rabbit anti-ecdysone antiserum has been used previously [16].

### Immunohistochemistry

Whole-mount immunohistochemistry was performed as described [21] with the following modifications: collagenase treatment was omitted, the incubation times with the primary and secondary antibody were 16 hr and 4 hr, respectively, and the anti-*Mab*PTTH mouse monoclonal antibody (D17) was used as the primary antibody.

### Collection and Processing of Hemolymph for Determination of the Ecdysteroid and PTTH Titers

Hemolymph was sampled at 12-hr intervals, immediately after lights-on and 12 hr after that point, and it was then processed as described previously [16].

### Determination of the Hemolymph Ecdysteroid Titers by Time-resolved Fluoroimmunoassay (TR-FIA)

To immobilize the 20-hydroxyecdysone (20E) on an EIA plate (Costar, 3590), its conjugate with ovalbumin was produced. Twenty-hydroxyecdysone was conjugated with ovalbumin using the ketone group at C-6 and carboxymethoxylamine, a bifunctional amino acid spacer, as described [23]. The wells of the EIA plates were incubated with 50 µl of 20E-ovalbumin conjugate solution (0.1 µg/ml in 50 mM Tris-HCl buffered saline (TBS)) overnight at 4°C, followed by blocking with 100 µl of 4% skimmed milk in TBS for 1 h at 25°C. After washing the wells with TBS containing 0.05% Tween-20, 50 µl of serially diluted 20E or the test samples were distributed to the wells, followed by the addition of 50 µl of 1∶100,000 diluted anti-ecdysone rabbit antiserum. The test samples, 20E and the antiserum were diluted with 0.5% bovine serum albumin in TBS. After overnight incubation at 4°C, the wells were washed, and the rabbit antibody bound to the immobilized 20E was quantified using the DELFIA system as described [16]. Because 20E was used as the standard hormone, the titers were expressed as 20E equivalents.

### Determination of Hemolymph PTTH Titers by TR-FIA

TR-FIA for *Mab*PTTH was essentially the same as that for *Bom*PTTH [16]. In the present assay, an anti-*Mab*PTTH mouse monoclonal antibody (D18) was immobilized on the EIA plate, and an anti-*Mab*PTTH rabbit antibody was used as the detection antibody. The brain extract was used as the standard hormone, as we had no purified native PTTH or correctly folded recombinant PTTH dimer. Brains of ND day-1 wandering larvae were dissected in insect saline and homogenized in 100 µl cold TBS containing 4-amidinophenylmethansulfonyl fluoride hydrochloride and using Bioruptor UCD-250, an ultrasonic homogenizer (Cosmo Bio, Tokyo, Japan). The homogenate was heated at 70°C for 5 min, cooled in an ice-water bath, and centrifuged at 15,000×*g* for 10 min. The supernatant was diluted with dilution buffer and used as the PTTH standard for TR-FIA, with one brain equivalent of PTTH defined as 1 unit.

### Quantitative RT-PCR for the Estimation of PTTH Gene Expression Levels

Quantitative RT-PCR was performed as described previously [24]. For the absolute quantification of mRNAs, serial dilutions of plasmids containing the cDNAs of *MabPTTH* and *RpL8* [25] were used for standards. After the molar amounts were calculated, the transcript levels of the *MabPTTH* were normalized with the *RpL8* levels in the same samples. The primers used in this analysis were as follows:

*MabPTTH* sense primer, 5′-GACGAGTACATGCTAGAAGACCAG-3′;

*MabPTTH* antisense primer, 5′-TCAGGTTGAATGGAATCCGTGTCG-3′;

*RpL8* sense primer, 5′-ATCAAGGGTGTCGTGAAGGACATC-3′;

*RpL8* antisense primer, 5′-CAGTAGACAAACTGGCCAGTGTAC -3′.

### Preparation and Injection of Crude PTTH

The brain extract was prepared as described above and concentrated by ultrafiltration over a 10 kDa cut-off membrane (Ultrafree, Millipore). The PTTH titer of the condensed brain extract was determined by TR-FIA, and the extract was then stored at −80°C. For PTTH absorption, the condensed brain extract was incubated with 1.4 µg per 10 units PTTH of anti-*Mab*PTTH or control rabbit antibody and 3.5 µl of Protein G-coupled agarose gel (Roche) for 1 hr. The supernatant after centrifugation was used as the PTTH-absorbed brain extract.

Prior to injection, the pupae were anesthetized by submersion in water for 1 hr. The brain extract was then injected into the vertex of the head. The wounds were sealed with melted paraffin.

### *In vitro* Culture of PGs

A small piece of thoracic integument to which the PG is attached (henceforth called the PG) was cut out in insect saline as previously described [26] and preincubated for 30 min in Grace’s Insect Medium. The PGs were cultured for 3 hr at 25°C in the medium (200 µl) containing varying doses of PTTH (the >10 kD brain extract prepared as above). The ecdysteroids released into the medium were measured by TR-FIA.

## Results

### Identification of *M. brassicae* PTTH (*Mab*PTTH)

**Figure 1:**
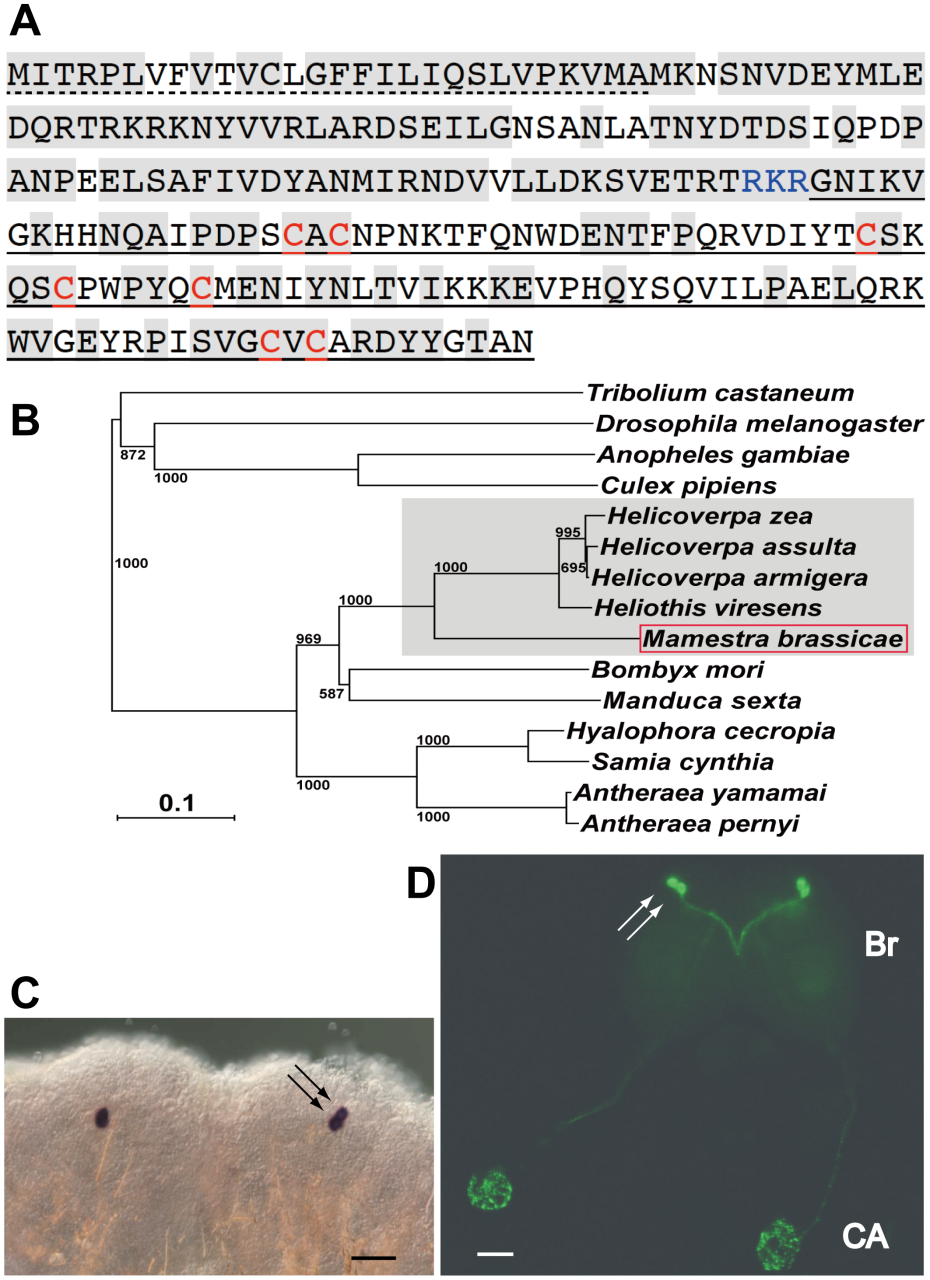
Identification of *M. brassicae* PTTH (*Mab*PTTH). (A) Amino-acid sequence of *Mab*PTTH precursor peptide. The dotted and straight underlines represent the predicted signal peptide and the PTTH monomer peptide, respectively. A predicted cleavage site is shown in blue letters. The seven cysteine residues conserved among the PTTHs are shown in red. The amino acid residues shown in shaded letters are conserved between the *M. Brassicae* and *H. armigera* PTTHs. (B) A phylogenetic tree showing the relationships between the PTTH precursors. The tree was generated using the ClustalW program (http://www.ebi.ac.uk/clustalw/). The shaded area denotes the Noctuidae family, and the red box shows *Mab*PTTH precursor. The scale bar indicates an evolutionary distance of 0.1 amino acid substitutions per position. The numbers on the branches denote bootstrap values per 1000 replications. (C) Whole-mount *in situ* hybridization analysis on a day-0 ND pupal brain with the antisense probe for the *MabPTTH* transcript. (D) Whole-mount immunohistochemistry on a day-0 sixth instar larval brain (Br)-corpora cardiaca-corpora allata (CA) complex with an anti-*Mab*PTTH mouse monoclonal antibody. The black and white arrows indicate the cell bodies of PTTH-producing neurons. Note that these neurons project their axons to the CA. Scale bars, 100 µm.

We isolated the entire sequence of the *Mab*PTTH cDNA by homology-based degenerate PCR and RACE strategies (see the Materials and Methods section for details). The predicted open reading frame encodes a 223-amino acid protein representing preproPTTH (Fig. 1A). This precursor protein exhibits a high homology to the corresponding proteins of other noctuid moths (Fig. 1B), with 69% identity to the *H. armigera* PTTH precursor [20]. The protein consists of a putative signal peptide (28 a.a.), a precursor domain (87 a.a.) and a PTTH monomer peptide (108 a.a.). Seven cysteine residues are present within the PTTH monomer at conserved locations, as in other known PTTHs [20]. *In situ* hybridization revealed that *MabPTTH* is expressed in two dorsolateral neurosecretory cells in each brain hemisphere (Fig. 1C), as are the PTTH genes of other lepidopteran insects [15], [22], [27].

The *Mab*PTTH cDNA was expressed in *E. coli,* but the product was a mixture of the misfolded monomer or oligomer of PTTH. Despite much effort with various methods, we were unable to obtain a correctly folded PTTH dimer, which is the biologically active form of PTTH. Therefore, the mixture was reduced and alkylated, then purified for use as an immunogen to produce the *Mab*PTTH antibodies that were necessary for developing the *Mab*PTTH immunoassay. All of the mouse monoclonal antibodies (D17 and D18) and the rabbit polyclonal antibody generated against this peptide were found to immunostain the same neurosecretory cells in the brain that were labeled by *in situ* hybridization (Fig. 1D). These neurosecretory cells project their axons to the corpora allata. These results indicate that in *M. brassicae,* PTTH is produced in two pairs of lateral brain neurosecretory cells and is released from the corpora allata, as in other lepidopteran insects [28].

### Developmental Changes in the Hemolymph Ecdysteroid Titer

The larvae were reared under either long-day (nondiapause-inductive) or short-day (diapause-inductive) conditions. In this study, the animals reared under the former and the latter conditions are designated as nondiapause-destined (ND) and diapause-destined (DD) animals, respectively.

**Figure 2:**
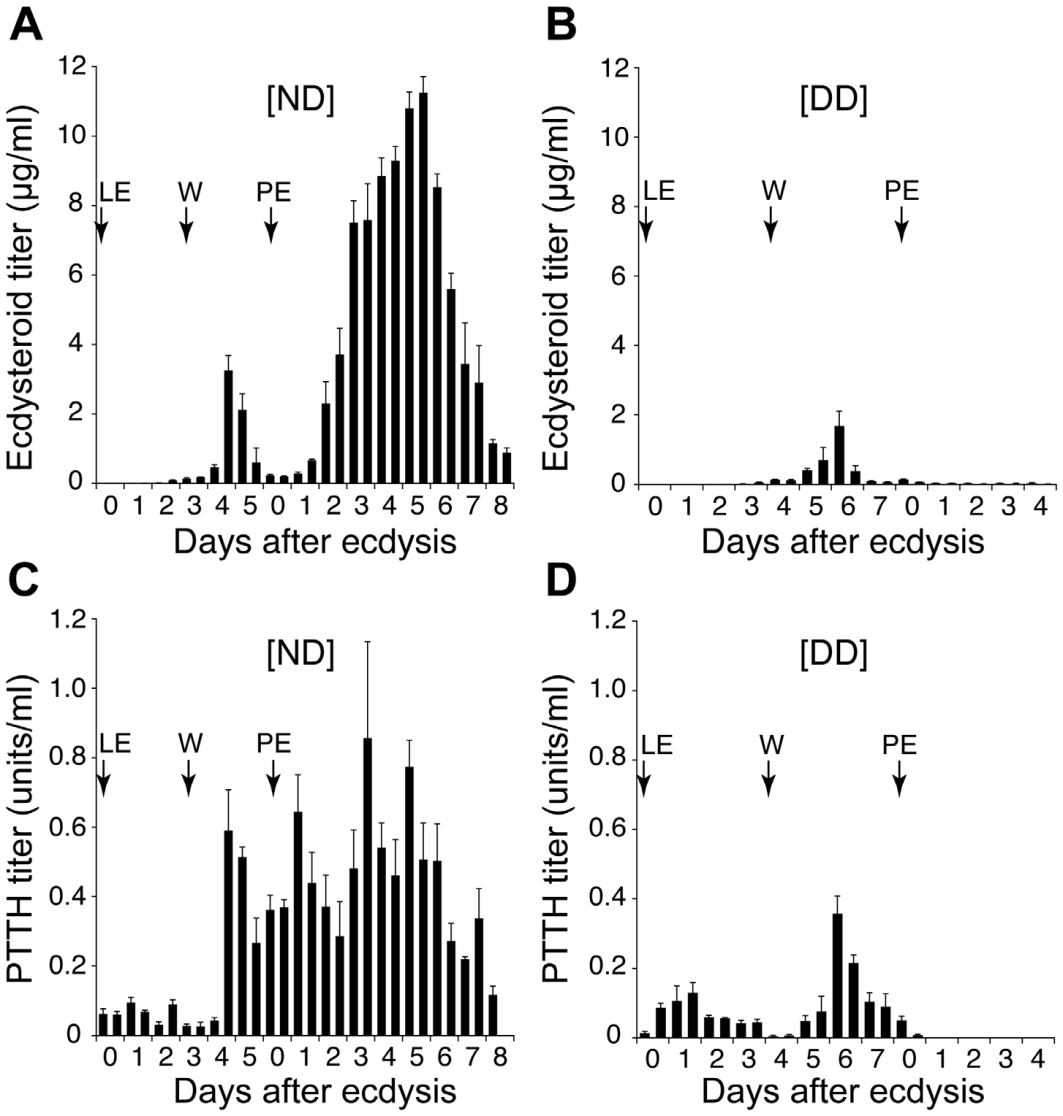
Developmental changes in ecdysteroid and PTTH titers in the hemolymph. (A,B) Ecdysteroid titers in the hemolymph of ND (A) and DD (B) animals. (C,D) PTTH titers in the hemolymph of ND (C) and DD (D) animals. The arrows indicate the times of the final larval ecdysis (LE), the onset of wandering (W) and pupal ecdysis (PE). The values shown are the mean (+SEM) of four independent determinations.

The shutdown of ecdysteroid secretion in diapausing pupae has been demonstrated in many insects. To confirm this shutdown for *M. brassicae*, hemolymph ecdysteroid titers were measured in both photoperiodic conditions (Fig. 2A,B). The overall pattern of the changes in the hemolymph ecdysteroid titer before pupal ecdysis was similar between the two types of animals, although the peak titer in the DD larvae was less than half the peak value in the ND larvae: this titer was slightly increased around the onset of wandering and peaked 2 days before pupation. In contrast, the titers after pupation differed remarkably between the ND and DD animals: the titer in the ND animals steeply increased 2 days after pupation, forming a large peak 3 days later, whereas no increase in the ecdysteroid titer was observed in the DD animals.

### Developmental Changes in the Hemolymph PTTH Titer

A very sensitive assay for *Mab*PTTH based on a TR-FIA protocol was developed, and the PTTH titers in the hemolymph of both ND and DD animals were determined (Fig. 2C,D). In the ND animals, the titer increased remarkably 2 days before pupal ecdysis. Although it dropped soon after that, the titer increased again at the time of pupal ecdysis, then fluctuated at high levels over the following week. The PTTH titer in the DD animals also increased before pupation, but the peak titer was only half the peak value of the ND animals. Following this peak, the titer gradually decreased and became undetectable 1 day after pupation. Interestingly, the first notable difference in the PTTH titer between the ND and the DD animals was observed during the early wandering stages, when the titers were significantly lower in the DD animals (two tailed t-test, p<0.01).

### Developmental Changes in PTTH mRNA and Protein Levels in the Brains of the ND and DD Animals

**Figure 3:**
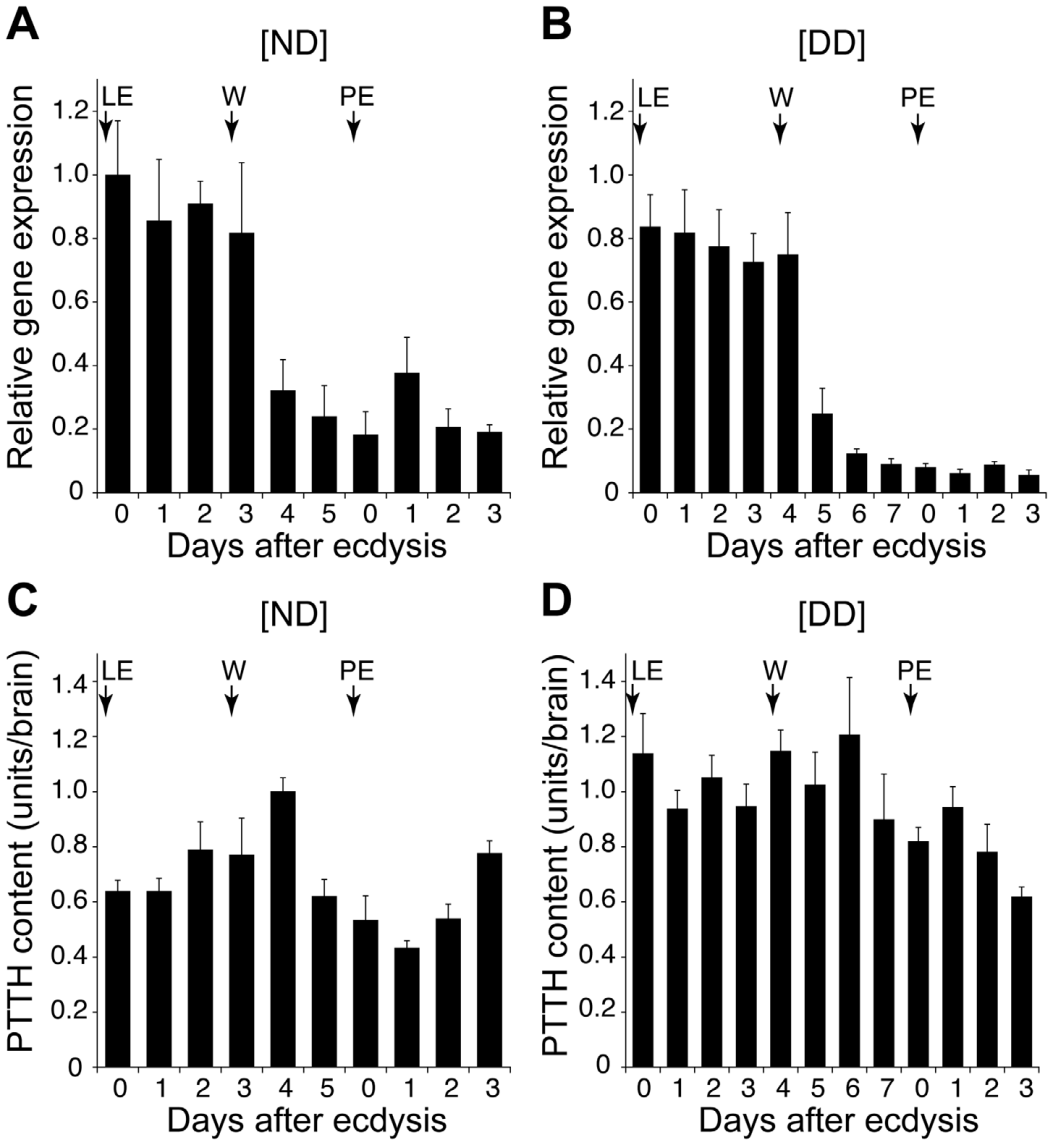
Developmental changes in PTTH gene expression and in the PTTH content in the brain. (A,B) PTTH gene expression in the brain of ND (A) and DD (B) animals, as determined by quantitative RT-PCR. The *PTTH* transcript levels were normalized with the *RpL8* transcript levels in the same samples and are indicated in relative values, with the highest value being 1.0. (C,D) PTTH content in the brains of ND (C) and DD (D) animals. Arrows indicate the times of the final larval ecdysis (LE), the onset of wandering (W) and pupal ecdysis (PE). The values shown are the mean (+SEM) of three (A,B) or five (C,D) independent determinations.

We next measured the PTTH mRNA and protein levels in the brain to determine what is responsible for the cessation of PTTH secretion in the DD animals. The mRNA level determined by quantitative RT-PCR was relatively high until the early wandering stage, when it decreased in both types of animals (Fig. 3A,B). Although the overall pattern of changes was similar, a notable difference was found between the ND and the DD animals after the decline of the mRNA level: a moderate level of PTTH mRNA was maintained until the early pupal stages in the ND animals, whereas the level was low in the DD animals, especially after pupation. This result indicates that PTTH gene expression is attenuated in the DD animals after the wandering stage.

However, the measurement of the brain PTTH content revealed that the reduced PTTH gene expression is not responsible for the cessation of PTTH secretion in the DD animals, because the PTTH content was actually higher in the DD animals than in the ND animals during the period from the early last instar to the second pupal day (Fig. 3C,D). This result clearly indicates that the cessation of PTTH secretion in the DD pupae is due not to a paucity of PTTH but rather to an attenuation of the secretory activity of the PTTH neurons.

### The Effect of PTTH Injection into the DD Pupae

**Figure 4:**
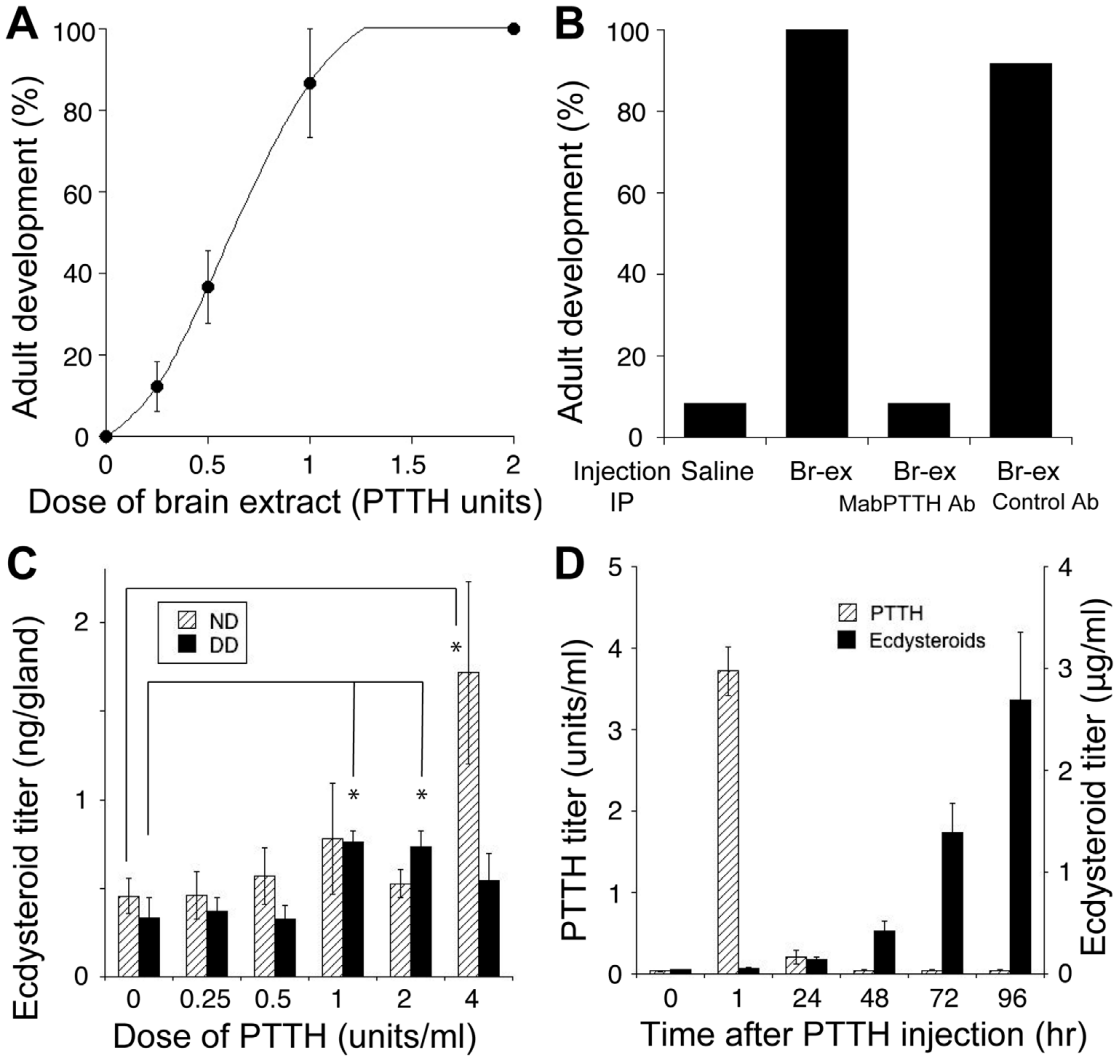
Effects of PTTH injections into day-0 DD pupae. (A) Dose-response of diapause escape by brain extract injection. Various doses of the brain extract were injected into five or six each of DD pupae within 16 hr of pupation, and the percentage of the animals that initiated adult development within a week was calculated. The values are the means ± SEM of three independent sets of experimental data. (B) Effects of PTTH absorption from the brain extract on the ability of the extract to avert diapause. The brain extract (Br-ex) was subjected to immunoprecipitation (IP) with an anti-*Mab*PTTH or a control antibody and then injected into day-0 DD pupae. As negative and positive controls, saline and the same dose (1 unit PTTH equivalent) of the brain extract were injected (n = 7). (C) Dose-dependent effects of PTTH on ecdysteroid secretion by the PGs *in vitro*. Day-0 pupal PGs from either ND or DD pupae were incubated *in vitro* for 3 hr in the absence or presence of serially diluted brain extract (PTTH), and the amounts of ecdysteroids secreted into the culture medium were determined. The values are the mean ± SEM (n = 6). Two-tailed *t*-test, *P<0.05. (D) Changes in PTTH and ecdysteroid titers after PTTH injection into day-0 DD pupae. One unit PTTH equivalent of the brain extract was injected, and the PTTH and ecdysteroid titers in the hemolymph were then monitored for 4 days. The values shown are the mean ± SEM (n = 5).

The cessation of PTTH secretion observed after pupation in the DD animals strongly suggested that PTTH serves as a switch between continuous development and pupal diapause. To test this interpretation, we injected a brain extract into the DD pupae immediately after ecdysis. The brain extract did induce adult development in a dose-dependent manner (Fig. 4A), demonstrating that the absence of some brain-derived factor in the hemolymph is the only cause of pupal diapause. To identify this brain factor, we removed PTTH from the brain extract by immunoprecipitation, then injected the extract into DD pupae of the same stage. The PTTH-absorbed brain extract failed to induce adult development (Fig. 4B), leading us to conclude that the diapause-averting factor in the brain is indeed PTTH.

### Activation of DD Pupal PG *in vitro* by PTTH

The successful induction of adult development in the DD pupae by PTTH injection suggested that the DD pupal PGs are as sensitive to PTTH as the ND pupal PGs, at least immediately after pupal ecdysis. To confirm this prediction, we investigated the dose-dependent effects of PTTH on the ecdysteroid secretion of day-0 pupal PGs *in vitro* (Fig. 4C). The basal activity of the PG, measured in the absence of PTTH, tended to be lower in the DD pupae than in the ND pupae. However, ecdysteroid secretion by the DD pupal PGs significantly increased in the presence of PTTH at 1 unit/ml or higher doses. By contrast, the ND pupal PGs did not significantly respond to PTTH at a concentration of 2 units/ml or less. Interestingly, at 4 units/ml of PTTH, the secretion was three times higher in the ND pupal PGs than in the DD pupal PGs. These results suggest that the DD pupal PG is sensitive enough to respond to PTTH, but the maximal rate of ecdysteroid secretion of this PG is less than that of the ND pupal PG.

### Changes in Hemolymph PTTH and Ecdysteroid Titers after PTTH Injection into the DD Pupae

Because a single injection of PTTH (brain extract) could block the initiation of diapause, we asked if the long-lasting release of PTTH observed in the ND pupae is essential for pupa-adult development. It was conceivable that the injected PTTH might have directly or indirectly stimulated the brain to release PTTH through a positive-feedback mechanism. Therefore, the changes in the PTTH and ecdysteroid titers in the hemolymph after the PTTH injection were monitored for 4 days (Fig. 4D). The PTTH titer increased immediately after injection but then declined rapidly, with no signs of re-elevation by day 4. In contrast, the ecdysteroid titer increased steadily following the injection. In the saline-injected control, no significant changes in either the PTTH or the ecdysteroid titer were observed.

## Discussion

Here, we have directly demonstrated, for the first time and using two different experimental lines, that the cessation of PTTH secretion after pupation leads to pupal diapause. The determination of the PTTH titer in hemolymph clearly showed that PTTH is absent in the hemolymph of the DD pupae, and the injection experiment provided evidence that the presence of PTTH in the hemolymph immediately after pupation is a necessary and sufficient condition for the pupa to continue developing into the adult. Taking these data together, we can therefore conclude that PTTH secretion serves as a switch between continuous development and pupal diapause, as first proposed by Carroll Williams over half a century ago.

During recent decades, various efforts have been made to verify the role of PTTH in the regulation of pupal diapause. Without suitable techniques to directly monitor PTTH secretion, the PTTH secretory activity of the brain was estimated using the PTTH content [9], [10], [17] or the PTTH gene expression levels [19], [20] in the brain. In the former cases, a similar or even higher level of PTTH activity was detected in the brain of early diapausing pupae compared to non-diapausing pupae. In the latter studies, lower PTTH gene expression was observed in the DD animals. These two observations are seemingly contradictory. This contradiction has previously been explained as species variation [2], as the above-mentioned experiments were conducted in different species. However, in the present study, both PTTH accumulation and *PTTH* downregulation are observed in the same species, suggesting that they are in fact general features of the late last instar larvae and of the early pupae of DD animals. The combination of data about the hemolymph PTTH titer and the brain PTTH content clearly indicates that the shutdown of PTTH release following pupal ecdysis in DD animals is caused not by a depletion of PTTH, but rather by a loss of the secretory activity of the PTTH neurons. This decline in secretory activity is accompanied by a downregulation of PTTH gene expression, suggesting that the cellular activity of PTTH neurons is wholly suppressed in the DD animals.

It is noteworthy that the decline of PTTH secretion in DD animals begins as early as the mid-last instar stage. The hemolymph PTTH titers measured during the early last instar stage were similar in both the ND and the DD animals, but the titer at its peak shortly before pupal ecdysis in the DD animals was only 60% of the titer of the ND animals. Because these differences in the hemolymph PTTH titer are first observed at the beginning of the wandering stage, a critical change(s) must have occurred in the brain of the DD animals by this time. In the previous studies, most efforts to analyze the molecular events leading to diapause have focused on the stage of animals around pupation, when diapause actually begins. However, our results strongly suggest that more attention should be paid to the wandering or earlier stages. The future studies addressing the molecular and/or cellular mechanisms of diapause induction should focus on these stages of development.

The successful induction of adult development by PTTH injection into day-0 DD pupae reveals two important points. First, the PGs may not differ between the DD and the ND pupae immediately after ecdysis. More specifically, the DD pupal PG, once stimulated by PTTH, may be able to secrete enough ecdysteroid to drive adult development. This possibility is supported by our *in vitro* observation that DD pupal PGs had similar basal secretory activity to ND pupal PGs and also had a high sensitivity to PTTH. The critical concentration of PTTH required to stimulate DD pupal PG was 1 unit (1 brain equivalent)/ml, which is comparable to or even lower than the levels that have been previously reported [10], [18], [29]. In this assay (Fig. 4C), the ND pupal PG was unexpectedly less sensitive than the DD pupal gland. This lower sensitivity is most likely because the ND pupal PG has already been exposed to PTTH (see Fig. 2C) and therefore has been somewhat desensitized to PTTH by the time of the assay. Interestingly, ecdysteroid secretion under high doses of PTTH (4 unit PTTH/ml) was much higher in the ND PGs than in the DD PGs (Fig. 4C), suggesting that the ND pupal PG has developed a higher capacity for ecdysteroid synthesis, while the DD pupal PG has not, because it has never been exposed to PTTH.

It is likely that PTTH is necessary not only for the immediate activation of the gland but also for the development of the gland’s capacity for ecdysteroid synthesis. This notion may be supported by our observation that the hemolymph ecdysteroid titer of the ND pupae starts to rise late on day 1 (Fig. 2A), while the PTTH titer is already high at the time of ecdysis (Fig. 2C). This time lag of hormone secretion might represent the time necessary for the inactivated PG to recover its high secretory activity under the stimulation by PTTH.

A comparison of the *in vitro* PG activity of ND and DD day-0 pupae has also been made in *M. sexta* [9]. In this study, although the basal secretory activity was almost the same, the DD pupal PGs secreted much less ecdysteroid than the ND pupal PGs after stimulation by PTTH, leading the authors to propose that diapause in this species is the result not only of the curtailment of PTTH release but also of a diapause-programmed refractory state of the PGs themselves. However, because a very high dose of PTTH (0.5 brain equivalents/25 µl, which corresponds to 20 brains/ml) was used in this study, the observed difference in ecdysteroid secretion upon PTTH stimulation could be interpreted in terms of differences in the gland’s capacity for ecdysteroid synthesis at the time of the assay.

Second, it is evident that a single PTTH stimulation event is sufficient to induce pupa-adult development. However, because the injected pupae had their own brains, it is possible that the injected PTTH stimulated the brain directly or indirectly via PG activation to release additional PTTH. This possibility was examined by monitoring the PTTH titer in the hemolymph after PTTH injection. No increase in PTTH was induced (Fig. 4D), indicating that the DD pupal brain remains inactive even in the developing adult. In contrast, in the ND pupae, the brain continually and actively secretes PTTH for several days following pupal ecdysis. If a brief stimulation by PTTH is sufficient for PG activation, then what is the significance of the prolonged release of PTTH? We have no definitive answer to this question because the PTTH-injected DD pupae normally develop into adults with almost the same time course as the ND pupae. PGs are presumed to degenerate during adult development, as demonstrated in *Drosophila melanogaster* [30], *M. sexta* [31], and other insects. Therefore, PTTH, a regulator of the PG, is no longer responsible for animal development. Thus, PTTH secretion might persist only because there is no need for its regulation. Alternatively, the excess release of PTTH might serve to ensure the prolonged activation of the PGs, even if the pupal PGs, once activated, are able to maintain and even enhance their activity by themselves.

In conclusion, we have shown unequivocally that the secretion of PTTH after pupal ecdysis serves as an endocrine switch between pupal diapause and adult development. It is evident that the cessation of PTTH secretion after pupal ecdysis is a necessary and sufficient condition for the pupae to undergo diapause. The reduced PTTH gene expression and increased PTTH accumulation in the brain of late final larvae are likely the common features in DD animals that reflect the attenuated cellular activity of the PTTH neurons. Our results highlight that the first sign of change in the PTTH neurons can be detected long before pupation. Future studies addressing the molecular mechanisms of diapause induction should therefore focus on this stage of development.
